# Unmanned Aerial Vehicle Assisted Post-Disaster Communication Coverage Optimization Based on Internet of Things Big Data Analysis

**DOI:** 10.3390/s23156795

**Published:** 2023-07-29

**Authors:** Biao Yang, Xuanrui Xiong, He Liu, Yumei Jia, Yunli Gao, Amr Tolba, Xingguo Zhang

**Affiliations:** 1 School of Communication and Information Engineering, Chongqing University of Posts and Telecommunications, Chongqing 400065, China; s210131282@stu.cqupt.edu.cn (B.Y.); s210131126@stu.cqupt.edu.cn (H.L.); s190101158@stu.cqupt.edu.cn (Y.J.); 2 School of Software, Dalian University of Technology, Dalian 116024, China; gylllll@mail.dlut.edu.cn; 3 Department of Computer Science, Community College, King Saud University, Riyadh 11437, Saudi Arabia; atolba@ksu.edu.sa; 4 Department of Mechanical Systems Engineering, Tokyo University of Agriculture and Technology, Nakacho, Koganei 184-8588, Japan; xgzhang@go.tuat.ac.jp

**Keywords:** Internet of Things big data analytics, unmanned aerial vehicle, wireless communication coverage

## Abstract

The rapid development of Internet of Things (IoT) communication devices has brought about significant convenience. However, simultaneously, the destruction of communication infrastructure in emergency situations often leads to communication disruptions and challenges in information dissemination, severely impacting rescue operations and the safety of the affected individuals. To address this challenge, IoT big data analytics and unmanned aerial vehicle (UAV) technologies have emerged as key elements in the solution. By analyzing large-scale sensor data, user behavior, and communication traffic, IoT big data analytics can provide real-time communication demand prediction and network optimization strategies, offering decision support for post-disaster communication reconstruction. Given the unique characteristics of post-disaster scenarios, this paper proposes a UAV-assisted communication coverage strategy based on IoT big data analytics. This strategy employs UAVs in a cruising manner to assist in communication by partitioning the target area into multiple cells, each satisfying the minimum data requirements for user communication. Depending on the distribution characteristics of users, flight–communication or hover-communication protocols are selectively employed to support communication. By optimizing the UAV’s flight speed and considering the coverage index, fairness index, and average energy efficiency of the mission’s target area, the Inner Spiral Cruise Communication Coverage (IS-CCC) algorithm is proposed to plan the UAV’s cruising trajectory and achieve UAV-based communication coverage. Simulation results demonstrate that this strategy can achieve energy-efficient cruising communication coverage in regions with complex user distributions, thereby reducing energy consumption in UAV-based communication.

## 1. Introduction

In the context of emergency communication scenarios, the severe destruction of traditional communication infrastructure significantly impacts the availability and effectiveness of communication, posing immense challenges to rescue operations and the affected individuals in disaster areas. However, in recent years, the rapid development of IoT big data analytics and UAV technology has brought about new solutions for emergency communication. With its powerful data processing and insights capabilities, IoT big data analytics, combined with the mobility and flexibility of UAVs, opens up new possibilities for emergency communication.

In post-disaster network reconstruction, IoT big data analytics plays several crucial roles. Firstly, it helps assess the extent of damage to communication infrastructure in affected areas and determines the priority of repairs. By analyzing real-time data and historical records, including sensor data, communication traffic, and user demands, an accurate evaluation of the communication needs in the damaged areas can be achieved, providing guidance for repair work. Secondly, IoT big data analytics can be used for planning and optimizing post-disaster communication networks. By analyzing the data traffic, user distribution, network topology, and resource allocation, optimal network planning schemes can be devised to ensure the maximization of communication coverage and efficient resource utilization. Additionally, IoT big data analytics can monitor network faults and anomalies, quickly detecting and rectifying issues, thereby enhancing the reliability and stability of the network. Most importantly, by integrating the IoT big data analytics with mobile devices such as UAVs, it becomes possible to bridge communication coverage gaps and rapidly establish temporary communication links. The flexibility and maneuverability of UAVs allow for the quick deployment and relocation in post-disaster areas, providing critical emergency communication support to affected regions. Equipped with communication devices, UAVs can serve as mobile communication relay stations, supplementing damaged infrastructure and maintaining the integrity of the communication network.

**Figure 1:**
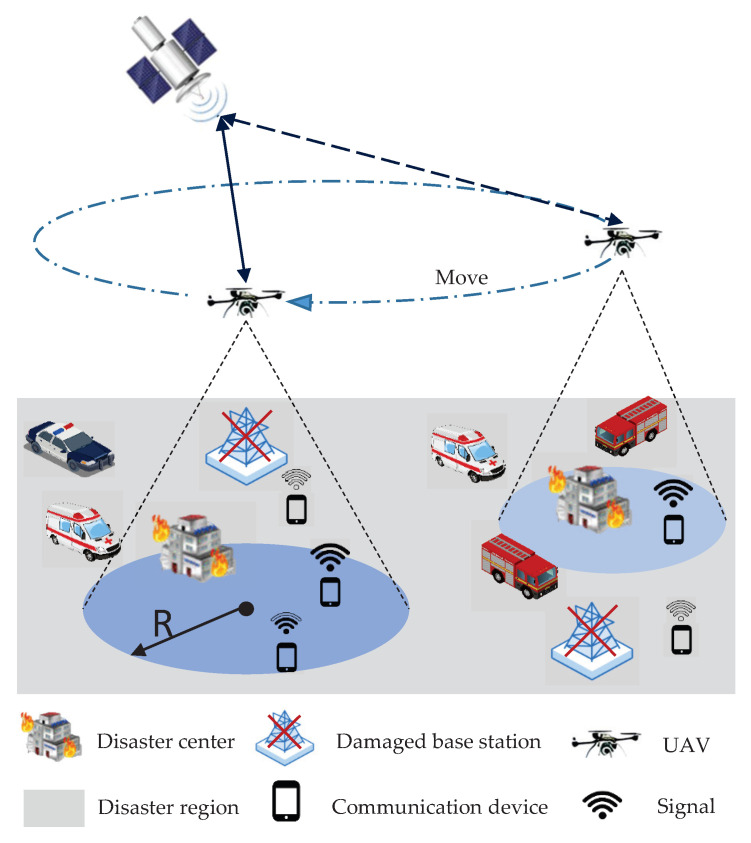
The system model for UAV-assisted emergency communication.

UAVs provide a versatile and flexible platform for assisting communication in emergency situations. Equipped with communication relay devices, UAVs can establish temporary wireless networks in areas where traditional infrastructure is damaged or unable to operate effectively, expanding the coverage and reach of communication services [1]. The combination of UAVs and IoT big data analytics enables UAVs to be strategically deployed, optimizing communication coverage, prioritizing communication needs, and adapting to evolving circumstances. The UAV-assisted emergency communication system model proposed in Figure 1. In the aftermath of disasters, when traditional communication base stations are damaged or unable to function properly, the flexibility and maneuverability of UAVs allow them to be rapidly deployed and repositioned in the affected areas, providing vital emergency communication support. Equipped with communication devices, UAVs can act as mobile communication relay stations, supplementing the damaged infrastructure and ensuring the integrity of communication networks. By carrying the communication relay equipment, UAVs can establish temporary communication links to provide emergency communication services to disaster-stricken areas [2]. Furthermore, UAVs can dynamically adjust their positions based on data collected from sensors, providing more comprehensive communication coverage and support [3].

Therefore, it is important to study the UAV communication coverage strategy in the emergency environment. A significant amount of research has focused on minimizing the task time and maximizing the energy efficiency for UAV-assisted communication [4,5,6]. However, most existing studies assume that the locations of ground users are known and their distribution is uniform. There is a lack of research on complex scenarios where post-disaster users have a non-uniform distribution and unknown locations. Furthermore, to our knowledge, limited attention has been given to the trade-offs between the limitations of UAV onboard batteries [7] and the maximization of communication coverage area in post-disaster scenarios. The challenges faced in establishing UAV-assisted communication coverage in emergency environments can be summarized as follows:

- To optimize UAV energy consumption, endurance, communication coverage, and quality, it is essential to quantify these parameters. However, there has been limited research on quantifying the UAV energy consumption and coverage range in emergency environments. Therefore, the challenge lies in how to achieve the maximum coverage range while minimizing UAV energy consumption, and jointly optimizing these two factors.
- In order to establish a UAV-assisted cruising communication coverage that is characterized by rapid deployment, high bandwidth, and adaptability to different environments, a multi-objective optimization problem needs to be formulated, which involves minimizing the cruise time and maximizing the communication coverage in user areas. However, achieving the minimum cruise time for maximizing communication coverage can be conflicting objectives. Therefore, addressing the multi-objective optimization problem and achieving a satisfactory balance between conflicting objectives is highly challenging.
- The objective of the UAV communication coverage strategy is to maximize the coverage rate index, the fairness coverage index, and minimize the UAV energy consumption for complex user distribution scenarios, while ensuring the connectivity of the UAV network at each time step. Achieving all these objectives is challenging.

The purpose of this paper is to address the complex post-disaster scenarios with uneven user distribution, and propose the use of UAVs for cruise coverage to restore communication. The main technical contributions of this paper are as follows:

- Due to the complexity of optimizing energy consumption for UAV-assisted communication coverage in post-disaster scenarios with uneven user distribution, a direct solution to this problem is challenging. In this paper, we propose a multi-objective optimization problem that aims to minimize UAV energy consumption while achieving maximum communication coverage. To effectively solve this problem, we decompose it into two sub-problems through the segmentation and integration of the optimization objectives.
- In the first sub-problem, the UAV assists ground users in communication through a combination of flight-communication mode and hover-communication mode. Then, the optimal flight speed for the UAV to meet the cruise communication coverage is analyzed, aiming to optimize the UAV’s flight speed and reduce its propulsion energy consumption.
- In the second sub-problem, due to the inability of the UAV to accurately obtain the locations of ground users, a bow-shaped or spiral-shaped cruise can be used to achieve the complete coverage of the target area in post-disaster scenarios. Considering that the energy consumption required for spiral-shaped coverage is less than that for bow-shaped coverage [8], we propose a UAV cruise communication coverage strategy, called the IS-CCC algorithm, to achieve the complete coverage of the post-disaster target area. This strategy allows the UAV to achieve the complete coverage of the target area with relatively low energy consumption.
- We compared and evaluated our proposed strategy with various coverage algorithms, considering the coverage rate index, fairness coverage index, and UAV energy consumption. Our aim was to demonstrate the effectiveness of our solution. The experimental results indicate that our approach successfully achieves a satisfactory balance between the UAV energy consumption and cruising communication coverage efficiency. Furthermore, it consumes less energy compared to other related algorithms.

The organization of the rest of this paper is as follows: Section 2 provides a review of related works. Section 3 introduces the system model. Section 4 presents the system optimization approach. Section 5 describes the system testing and results analysis. Finally, Section 6 concludes the paper.

## 2. Related Works

In this section, we reviewed the relevant work of IoT big data analytics and the use of UAVs in post-disaster communication networks.

In this era of the IoT, IoT big data analytics plays a crucial role in various fields such as smart city transportation [9] and post-disaster network reconstruction [10]. The IoT connects various physical devices, sensors, and terminal devices, generating massive amounts of data [11]. Through IoT big data analytics, valuable information and insights can be extracted from these data, bringing many applications and benefits to various industries. Firstly, IoT big data analytics can assess and predict post-disaster communication needs by collecting and analyzing sensor data, user demands, and communication traffic in the post-disaster communication network. By deploying a central control system to allocate computing and caching resources to mobile users [12], decision makers can understand the communication requirements of the affected areas, including the number of people, communication traffic, and types of communication services. This enables them to plan and effectively allocate resources. Secondly, IoT big data can help in planning and configuring the infrastructure and resources of post-disaster communication networks. By analyzing the geographical data, population distribution, and communication demands, the locations, quantities, and coverage areas of communication base stations, as well as the types and capacities of communication equipment, can be determined. This optimization of network layout ensures that communication services meet the needs of post-disaster rescue efforts. In summary, IoT big data analytics plays an important role in the planning and optimization of post-disaster communication networks. The related work covers areas such as post-disaster communication needs assessment, network planning and design, topology optimization, fault detection, and recovery, and resource scheduling and management. These efforts provide strong support for achieving efficient and reliable post-disaster communication networks.

UAVs have been widely used in wireless communication. The combination of UAVs with mobile edge computing (MEC) is considered a promising technology with applications in various fields such as medical monitoring [13], intelligent agriculture [14], intelligent transportation [15], emergency communication [16], etc. The development of edge computing and content caching in wireless networks can provide high-quality communication services to customers [17]. It has become a key enabling technology in addressing a series of problems caused by the increasing number of interconnected devices and large-scale data transmission [18]. For example, Wang et al. [19] proposed an online scheduling scheme for edge servers based on UAVs, which merges tasks geographically into several hotspots and schedules UAVs to appropriate hover positions, achieving less UAV scheduling for more user demands. Ning et al. [20] designed a 5G-enabled UAV to community offloading system, which maximizes the throughput.

The application of UAVs in emergency communication systems for communication coverage needs to fulfill specific requirements, including rapid deployment, easy operation, low cost, high capacity, and wide coverage. Using UAVs as aerial communication relay platforms can effectively improve the coverage and capacity of the network, with coverage, capacity, and latency meeting the basic requirements of emergency communication [21]. UAV communication coverage involves UAVs carrying long-term evolution communication payloads moving over disaster areas or hovering in a certain position to serve as aerial base stations for ground user communication. Meanwhile, blockchain has been used to ensure the security of mobile data transactions [22], and has been widely applied in civilian [23,24] and military applications [25]. Aggarwal et al. [26] proposed a blockchain-based security solution and 6G-enabled network connectivity in UAV communications to address the security concerns during the communication coverage of UAVs. This solution offers enhanced security measures to mitigate the potential risks and vulnerabilities in UAV communication systems. When using deep reinforcement learning techniques to assist in the deployment and communication of multiple UAVs, it has been extensively studied by many scholars. For example, in order to effectively control the network topology, Liu et al. [27] adopted the deep deterministic policy gradient to adjust the connection with other UAVs to minimize the learning time and improve the performance of the system. In order to improve the performance of wireless networks, Zhong et al. [28] adopted an effective framework based on the deep Q-Network to manage the dynamic motion of UAVs and maximize the total data transmission rate of ground users. Liu et al. [29], in order to solve the deployment and motion design of UAV network, the deployment algorithm based on Q-Learning can show a fast convergence rate after a few iterations to obtain its own three-dimensional position and enable the rapid deployment of UAV.

The research on UAV communication coverage in emergency areas is mainly concentrated in several aspects, including resource allocation [30,31], height optimization [32], trajectory optimization [33,34], and multi-UAV collaborative coverage [35]. Liu et al. [36] investigated post-disaster multi-UAV collaborative control strategies and proposed a distributed multi-UAV control solution based on deep reinforcement learning. Although the above research has explored post-disaster UAV-assisted communication technologies from multiple perspectives, UAVs also have certain limitations in post-disaster networks. First, consider that the UAV flight time and range are limited by battery life and energy, which may limit the sustainability and efficiency of UAVs in large-scale or prolonged rescue missions; Secondly, high wind speed and a complex environment may challenge the stability and control ability of UAV, limiting its flight ability under certain conditions; Finally, the simultaneous deployment of UAVs in a post-disaster network can also involve coordination and resource allocation issues, requiring effective deployment strategies and team collaboration. While UAVs have many advantages in post-disaster networks, such as rapid response, efficient data acquisition, and rescue capabilities, understanding these limitations is critical to optimizing their applications and addressing related issues. In addressing these limitations, we can consider technological improvements, collaboration, and rational use strategies to improve the effectiveness of UAVs in post-disaster networks.

## 3. System Model

### 3.1. UAV Power Consumption Model

In the process of UAV-assisted wireless communication, the energy consumption of UAVs can be attributed to two main factors: communication-related energy consumption and propulsion energy consumption [37]. Communication-related energy consumption encompasses the energy required for UAVs to engage in communication with ground users. This includes energy consumption associated with communication circuits, signal processing, signal radiation, and reception, among other factors. Furthermore, UAVs also necessitate propulsion energy consumption to sustain a high-altitude flight and facilitate movement. Presently, fixed-wing UAVs and rotary-wing UAVs are among the commonly used types of UAVs. Indeed, rotary-wing UAVs offer the advantage of vertical takeoff and landing, as well as the ability to hover at a fixed position, which enables them to provide stable network coverage for ground areas. In this section, the focus is primarily on analyzing the energy consumption model of rotary-wing UAVs. This model encompasses both communication-related energy consumption and propulsion energy consumption.

#### 3.1.1. Propulsion Energy Consumption

Generally, the propulsion energy consumption of UAVs is primarily determined by the flight speed and acceleration of the UAVs. This refers to the power necessary for propelling the UAV during its flight. In this study, the focus is on UAVs covering the target area in a stable and uniform manner at a constant speed. Therefore, the additional energy consumption caused by UAV acceleration is disregarded. It is worth noting that the time taken for UAV acceleration and deceleration during operation constitutes a small fraction of the overall mission time. Hence, it is reasonable to neglect UAV acceleration for typical communication application scenarios. When a rotary-wing UAV flies at a constant speed *v*, the propulsion power consumption of the UAV can be modeled as Equation (1) [37]:

(1) $$P\left(vs.\right)=P_{0}\left(1+\frac{3v^{2}}{U_{tip}^{2}}\right)+P_{i}{\left(\sqrt{1+\frac{v^{4}}{4v_{0}^{4}}}-\frac{v^{2}}{2v_{0}^{2}}\right)}^{\frac{1}{2}}+\frac{1}{2}d_{0}\rho sAv^{3}$$

where $P_{0}=\rho sA\Omega^{3}R^{3}\delta /8\;$ represents the blade profile power, $P_{i}=\left(1+k\right)W^{\frac{3}{2}}/\sqrt{2\rho A}\;$ represents the induced power when the UAV is in hover state; and $P_{0}$ and $P_{i}$ are constants. $d_{0}$, ρ, *s*, *A*, $U_{tip}$, and $v_{0}$ are constants related to aerodynamics and aircraft design, with specific parameter values shown in Table 1. From Equation (1), it can be observed that the propulsion power consumption of a rotary-wing UAV mainly consists of three parts: blade profile power, induced power, and parasite power. The parasite power increases with the cube of the UAV’s flight speed to overcome the body drag of the UAV.

When the rotary-wing UAV is in a hovering state, the flight speed v=0 can be set to zero, and Equation (1) can be simplified as:

(2) $$P_{h}=P_{0}+P_{i}$$

Based on Equation (2) analysis, it can be inferred that the power of a rotary-wing UAV during hovering is a finite value, mainly determined by factors such as the weight of the UAV, air density, and the area of the rotor disc. When the flight trajectory q(t) of the UAV is given, the energy consumption for the propulsion of the UAV can be expressed as [38]:

(3) $$E_{1}\left(T_{t},\left\{q\left(t\right)\right\}\right)=\int_{0}^{T_{t}}P\left(||v\left(t\right)\left|\right|\right)dt$$

where $T_{t}$ represents the duration of the UAV’s flight mission, and $||v\left(t\right)\left|\right|$ represents the instantaneous velocity of the UAV at any given time *t*.

#### 3.1.2. Communication-Related Energy Consumption

When the UAV provides communication access to ground users, it incurs the energy consumption associated with communication. It is worth noting that the energy consumption related to communication is typically much lower compared to the propulsion energy consumption, usually in the order of a few watts, while the propulsion energy consumption can be in the hundreds of watts. Assuming the UAV’s communication power is $P_{c}$ (not necessarily a constant), the energy consumption related to communication can be represented as:  

(4) $$E_{2}\left(T_{t},P_{c}\right)=\int_{0}^{T_{t}}P_{c}\left(t\right)dt$$

Therefore, with the energy consumption models for both propulsion and communication-related energy, the total energy consumption of the UAV can be represented as:

(5) $$E\left(T_{t},\left\{q\left(t\right)\right\},P_{c}\right)=E_{1}+E_{2}$$

### 3.2. UAV Cruise Communication Coverage Model

#### 3.2.1. Communication Energy Consumption Formulization

**Figure 2:**
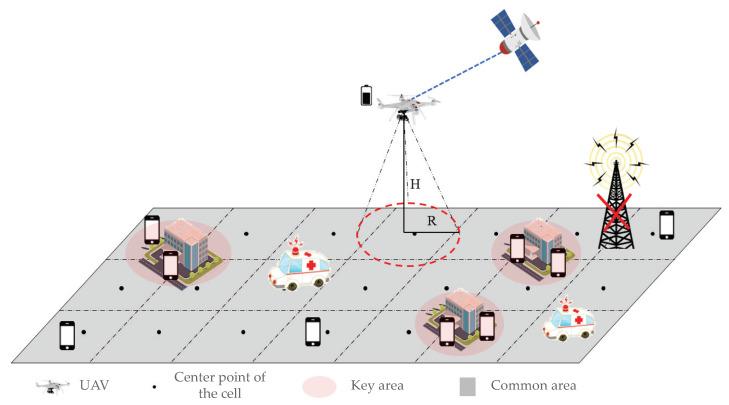
UAVs cruise communication coverage model.

When a natural disaster causes a ground base station to fail, the UAVs can be used as an aerial base station to quickly establish an emergency communication network to restore communication for ground users. Figure 2 shows a proposed scenario of a UAV cruising for communication coverage in complex post-disaster scenes where users are distributed in various locations, with the majority concentrated in safe places such as schools and shelters, and a few users sparsely distributed in arbitrary positions within the target area. Therefore, only the positions of key areas such as safe places, denoted as g∈G, can be obtained, while the location information of other users cannot be acquired. Assuming that the target area is a rectangular disaster area with a size of L×M (m), the rotary-wing UAV is used to cruise at a fixed flying altitude of H(m) as an airborne temporary base station, covering the target area. The coverage area of the UAV on the ground is a circular region with a radius of R(m), and the UAV establishes communication links with satellites to meet communication needs. The UAV can flexibly adjust the transmit power to adjust the coverage range on the ground based on demand.

Considering that there are more users with higher communication demand in key areas such as safe places, the *v* adopts a hover-communication protocol to assist user communication when cruising over key areas with dense user distribution, while using a flight–communication protocol to assist user communication in other areas. Due to the complexity of the task of UAV cruising and communication coverage in the target area, the target area is divided into grids, and the UAV covers each grid by hovering over the center point of each grid cell to achieve communication coverage in the target area.

In this scenario, we assume that high buildings are severely damaged after a disaster, and the probability of non-line-of-sight components in the received signal is much lower than the probability of line-of-sight components. Therefore, an air-to-ground channel model is used for signal transmission, and the influence of small-scale fading is neglected. In this case, the air-to-ground channel can be approximated as a free space channel model, and its channel power gain can be expressed as:

(6) $$h\left(t\right)=\beta d^{-2}\left(t\right)$$

where β represents the channel power gain at a reference distance of 1 m, and $d^{-2}\left(t\right)$ represents the distance between the UAV and a ground user at time *t*. Assuming that $T_{t}$ represents the total mission time for the UAV to cruise and provides the communication coverage in the target area, then at any time $t\in [0,T_{t}]$, the distance between the UAV and any ground user *n*, where *n* belongs to the set of all ground users n∈N, can be expressed as:  

(7) $$d_{n}\left(t\right)=\sqrt{{∥q\left(t\right)-w_{n}∥}^{2}+H^{2}}$$

where $w_{n}\in R^{2\times 1}$ represents the horizontal position of any ground user, and represents the projection of the UAV’s trajectory onto the horizontal ground.

According to the analysis of the system model, it is known that the coverage area of the UAV on the ground changes over time. This means that establishing a complete connection and completing data transmission between ground users and the UAV requires a certain duration of continuous coverage time, denoted as $t_{c}$. Therefore, only when the association time between ground users and the UAV is greater than $t_{c}$ can the connection between them be successfully established.

Assuming that the total available bandwidth *B* is uniformly divided into $L=\left\{1,2,\ldots ,L\right\}$ orthogonal channels during the process of communication data transmission, the achievable rate of user *z* at time *t* when it successfully establishes communication can be expressed as follows:

(8) $$R_{z}^{l}\left(t\right)=B_{l}\log_{2}\left(1+\frac{P_{U}\beta }{d^{2}\left(t\right)N_{0}}\right)$$

where $P_{U}$ represents the transmission power of the UAV, $N_{0}$ represents the noise power of the boundary user, $B_{l}$ represents the bandwidth of subchannel *l*, and l∈L. Only when the total amount of data received by boundary user *z* within the duration of continuous coverage $t_{c}$ is greater than or equal to the minimum data amount Q bits can it be considered that the UAV meets the communication requirements of the ground users, as expressed in Equation (9).  

(9) $$\int_{0}^{t_{c}}R_{z}^{l}\left(t\right)dt\geq Q,\forall n$$

When the UAV is flying horizontally at a constant speed *v*, the propulsion power consumed can be calculated using Equation (1) as P(v). Considering the weather and other reasons [39], when the UAV is hovering over key areas with a high concentration of users using a hover-communication protocol for coverage, the hover power of the UAV can be calculated using Equation (2) as $P_{h}$. Therefore, the energy consumption of UAV’s cruising communication coverage can be approximated as:

(10) $$E\approx P\left(vs.\right)\frac{q\left(t\right)}{v}+P_{h} [T_{t}-\frac{q\left(t\right)}{v}]+P_{c}T_{t}+\frac{(\kappa +\gamma )H}{1000}$$

where $T_{t}$ represents the total mission time of the UAV’s cruising coverage over the target area, $q\left(t\right)/v\;$ represents the UAV’s flight time, $T_{t}-q\left(t\right)/v\;$ represents the UAV’s hover time, and $P_{c}$ represents the communication power related to communication during the UAV’s cruising coverage process, κ and γ are specific attenuations caused by weather and gases.

The research objective of this part is to optimize the energy consumption of UAV’s cruising communication coverage by optimizing the UAV’s flight speed and planning UAV’s flight trajectory based on the cruise coverage of the target area. The research problem can be modeled as follows:

(11) $$\begin{array}{cc} & \min_{v,\left\{q\left(t\right)\right\}}\;E\left(v,\left\{q\left(t\right)\right\}\right)\\ & s.t.\int_{0}^{t_{c}}B_{l}\log_{2}\left(1+\frac{P_{U}\beta }{d^{2}\left(t\right)N_{0}}\right)dt\geq Q,\forall n,\forall t\in [0,T_{t}],\\ & \;\;\;\;\;\;\;\;\;\;0\leq v\leq V_{\max},\\ & \;\;\;\;\;\;\;\;\;\;q\left(0\right)=q_{I},q\left(T_{t}\right)=q_{F},\\ & \;\;\;\;\;\;\;\;\;\;h_{\min}\leq H\leq h_{\max},\\ & \;\;\;\;\;\;\;\;\;\;E_{f}+E_{h}+E_{c}\leq E_{\max}. \end{array}$$

where $q_{I},q_{F}\in R^{2\times 1}$, respectively, represent the initial and final positions of the UAV projected onto the ground, while $E_{f}$ and $E_{h}$ represent the energy consumption for UAV flight and hover, respectively. $E_{c}$ represents the energy consumption associated with communication-related tasks during UAV flight or hover.

#### 3.2.2. Coverage Evaluation Formulization

As the task objective is to meet communication coverage in complex post-disaster user distribution scenarios, it is necessary to define the coverage indicators of the target area cells. First is the coverage index, which is used to measure the number of times a cell is covered in a single task. For any cell at any time *t*, the coverage index is expressed as:

(12) $$c_{t}=\left\{\begin{array}{cc} & c_{t}\left(vs.\right)=\frac{\sum_{v=1}^{V}T_{t}\left(vs.\right)}{VT},\forall v\in 1,\cdots ,V\\ & c_{t}\left(u\right)=\frac{\sum_{u=1}^{U}T_{t}\left(u\right)}{U\left(T-t_{h}\right)},\forall u\in 1,\cdots ,U \end{array}\right.$$

where $T_{t}\left(u\right)$ or $T_{t}\left(vs.\right)$ represents the number of times the key unit *u* or ordinary unit *v* is covered at the current time *t*, and *T* represents the total mission time. It should be noted that the continuous coverage of key units is assumed to be a single coverage, and the coverage rate index only considers the coverage times in time. Since the goal of UAV collaborative communication coverage is to maximize the total coverage of the target area, simply improving the coverage rate index may result in unfair coverage of all units, that is, in most time steps, a few units are continuously covered while the remaining units are never covered. Therefore, it is necessary to solve the fairness problem of unit coverage, which is a spatial index used to measure whether all units are fairly covered in space. The corresponding fairness coverage index for a given target area control strategy is represented as follows:   

(13) $$f_{t}=\frac{{ [c_{t}\left(vs.\right)+c_{t}\left(u\right)]}^{2}}{K [c_{t}{\left(vs.\right)}^{2}+c_{t}{\left(u\right)}^{2}]}$$

As analyzed from Equation (13), the larger the fairness coverage index $f_{t}$, the more equitable the coverage of the target cells, where ft∈11KK,1. Furthermore, the UAV flight inherently consumes energy, and it is crucial to minimize this energy consumption to extend the network’s overall lifetime.

Using the coverage rate index $c_{T}$, fairness coverage index $f_{T}$, and average energy efficiency $\eta_{T}$ as evaluation metrics, the average energy efficiency $\eta_{T}$ can be expressed as:

(14) $$\eta_{T}=\frac{c_{T}\times f_{T}}{{\bar{e}}_{T}}$$

where ${\bar{e}}_{T}$ represents the average energy consumption of the UAV during the test period. This value is normalized by the maximum possible energy consumption of the UAV during the same test period.

In summary, the goal of the UAV communication coverage strategy in scenarios with complex user distribution is to maximize the coverage index, fairness coverage index, and minimize UAV energy consumption, while ensuring network connectivity at each time step. Indeed, achieving all these objectives can be quite challenging. On the one hand, UAVs need to patrol continuously to provide effective and equitable communication coverage for target cells, which involves effectively distributing their presence in both time and space domains. On the other hand, minimizing the total energy consumption of UAVs and ensuring network connectivity requires reducing the movement of UAVs while maintaining stable communication connections. Reducing the movement of UAVs can save energy, while maintaining communication connections is crucial for ensuring network connectivity. Balancing these requirements is a complex task. Therefore, a good UAV control strategy should balance this issue well.

## 4. Optimization of Energy Consumption for UAV Cruise Communication Coverage

The optimization problem of energy consumption for UAV cruise communication coverage in complex areas with post-disaster user distribution is too complicated to be directly solved. Therefore, this problem is divided into two sub-problems. Firstly, the optimal flight speed for the UAV to meet cruise communication coverage is calculated, ensuring that the UAV achieves energy optimization by using the minimum power to satisfy the coverage requirements. Secondly, the flight trajectory for the UAV to cruise and cover the target area is planned, aiming to achieve maximum coverage with the least cruise time, thus optimizing the energy consumption of the UAV. By solving these two sub-problems, energy optimization for post-disaster UAV cruise communication coverage is achieved.

### 4.1. Cruising Speed Optimization

First, let us solve sub-problem one, which involves analyzing the optimal flight speed for the UAV to meet cruise communication coverage. As mentioned earlier, the UAV adopts a combination of flight-communication protocol and hover-communication protocol to assist ground users in communication. Therefore, by optimizing the flight speed of the UAV, the energy consumption for propulsion can be reduced. When the UAV is engaged in assisting communication, it will be equipped with a directional antenna with an adjustable wave speed. The half-beamwidth of the directional antenna is denoted as ω, where 0<ωmin≤ω≤ωmax<ππ22, $\omega_{\min}$ and $\omega_{\max}$ are determined by the technology used to adjust the actual beam width of the antenna [40].

**Figure 3:**
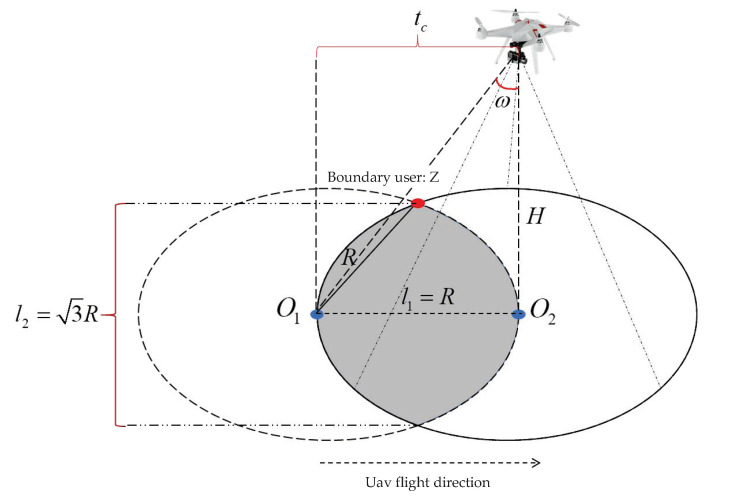
Schematic diagram of UAV flight speed analysis.

As shown in Figure 3. Assume that the UAV departs from point $O_{1}$ at time $t_{0}=0$ and reaches point $O_{2}$ at time $t_{1}=vt_{c}$. Since the width of the shadow region varies with changes in the UAV’s coverage radius and flight speed, in order to analyze the cruise speed of the UAV while satisfying ground user communication, we define the width of the shadow region as $O_{1}O_{2}=R$, which represents the flying distance that satisfies $d_{O_{1}O_{2}}=R$, the total coverage time of users within the shadow region as tc=dO1O2dO1O2vv=RRvv. From geometric analysis, it can be determined that the width of the shadow region is denoted as $l_{1}=d_{O_{1}O_{2}}=R$, and the length of the shadow region as $l_{2}=\sqrt{3}R$. There is a relationship equation ω=arctanRRHH between the half-beamwidth of the antenna, coverage radius, and the height of the UAV.

As the UAV’s cruise communication coverage time progresses, the distance between the UAV and the users constantly changes. The distance between the UAV and the boundary user *z* can be represented by Equation (7).

(15) $$d_{z}\left(t\right)=\sqrt{{\left(\frac{\sqrt{3}R}{2}\right)}^{2}+{\left(\frac{R}{2}-vt\right)}^{2}+H^{2}}$$

At any given moment *t*, the received signal-to-noise ratio (SNR) of the boundary user *z* can be expressed as:  

(16) $$SNR_{z}\left(t\right)=\frac{P_{U}\beta G_{0}}{d_{z}^{2}\left(t\right)N_{0}\omega^{2}}$$

where G0G0ω2ω2 represents the antenna gain within the main lobe of the directional antenna of the UAV. In order for boundary user *z* to establish communication, the total communication data volume of the boundary user must be greater than or equal to *Q* bits within the continuous coverage time period $t_{c}$, which can be expressed as:

(17) $$\int_{0}^{t_{c}}B_{l}\log_{2}\left(1+\frac{P_{U}\beta G_{0}}{d_{z}^{2}\left(t\right)N_{0}\omega^{2}}\right)dt\geq Q$$

To optimize the energy consumption of the UAV’s cruise communication coverage in the target area, substituting ω=arctanRRHH and Equation (15) into Equation (17), and letting α=PUβG0PUβG0N0arctanRRHH2N0arctanRRHH2, and then making a variable substitution for x=vt, the expression can be obtained as follows:

(18) $$\begin{array}{cc} & \frac{B_{l}}{v\ln2}\int_{0}^{R} [\ln\left({\left(\frac{\sqrt{3}R}{2}\right)}^{2}+{\left(\frac{R}{2}-x\right)}^{2}+H^{2}+\alpha \right)\\ & \left.-\ln\left({\left(\frac{\sqrt{3}R}{2}\right)}^{2}+{\left(\frac{R}{2}-x\right)}^{2}+H^{2}\right)\right]dx\geq Q \end{array}$$

further calculating Equation (18) and letting y=RR22−x, the expression (16) can be obtained as follows:

(19) $$\frac{B_{l}}{v\ln2}\int_{-R}^{0} [\ln\left({\left(\frac{\sqrt{3}R}{2}\right)}^{2}+y^{2}+H^{2}+\alpha \right)-\ln\left({\left(\frac{\sqrt{3}R}{2}\right)}^{2}+y^{2}+H^{2}\right)]dy\geq Q$$

continuing the calculation of Equation (19), defining a=3R3R222+H2+α and b=3R3R222+H2, and substituting *a* and *b* into the expression and integrating, the resulting expression is as follows:

(20) $$\frac{B_{l}}{v\ln2} [R\ln\left(R^{2}+a^{2}\right)+2a\arctan\left(\frac{R}{a}\right)-R\ln\left(R^{2}+b^{2}\right)+2b\arctan\left(\frac{R}{b}\right)]\geq Q$$

by simplifying Equation (20), the cruising speed of the UAV that satisfies communication with boundary users can be expressed as follows:

(21) $$v\leq \frac{B_{l}}{Q\ln2} [R\ln\left(R^{2}+a^{2}\right)+2a\arctan\left(\frac{R}{a}\right)-R\ln\left(R^{2}+b^{2}\right)+2b\arctan\left(\frac{R}{b}\right)]$$

The arctangent function can be approximated as arctanx≈xx1+0.28x21+0.28x2. Substituting this function, as well as the values of *a* and *b*, into the formula, the cruising speed of the UAV that satisfies the communication coverage can be calculated as $v^{\prime }$.

(22) $$\begin{array}{cc} & v\;\;{\;}^{\prime }\;\;\;\leq \frac{B_{l}}{Q\ln2}\left\{R\ln\left(\frac{1.75R^{2}+H^{2}+\alpha }{1.75R^{2}+H^{2}}\right)\right.\\ & +\frac{R\left(-1.78R^{2}\alpha^{2}-2H^{2}\alpha^{2}-\alpha^{4}\right)}{ [\left(0.75R^{2}+H^{2}+\alpha \right)\left(0.75R^{2}+H^{2}\right)]}\\ & \left.\times \frac{1}{\left(0.75R^{2}+H^{2}+\alpha \right)\left(0.75R^{2}+H^{2}\right)+0.28R^{2}\left(1.5R^{2}+2H^{2}+\alpha^{2}\right)+0.784R^{2}}\right\} \end{array}$$

### 4.2. Trajectory Optimization

In this subsection, the sub-problem of the UAV cruising communication coverage strategy is solved, which involves planning the UAV’s flight trajectory. Due to the UAV’s inability to accurately obtain the ground users’ locations, in order to achieve communication coverage for all ground users in the post-disaster scenario, a circular or spiral cruising pattern can be adopted to achieve the full coverage of the target area. For rectangular areas, considering that the energy consumption required for spiral coverage is lower than that for circular coverage, a spiral pattern is adopted for covering the target area.

When the UAV achieves normal communication with the boundary users, the width of the shadow area covered by the UAV is denoted as $l_{1}=R$, and the length of the shadow area is denoted as $l_{2}=\sqrt{3}R$. Due to the fact that the coverage area of the UAV on the ground is in the form of a strip during flight communication coverage, the target area is rasterized using cells with a side length of $\sqrt{3}R$. The geometric center points and centroid regions *g* of each cell are obtained as waypoints for the UAV’s planned path. By cruising through all the waypoints, the UAV can achieve communication coverage over the target area. Rasterizing the target area into cells allows for the transformation of the communication coverage problem into a UAV trajectory planning problem with known waypoints.

To achieve the full coverage of the target area, an IS-CCC algorithm is proposed. In this algorithm, the UAV cruises in an inner spiral pattern to cover all the geometric center points $q_{N}$ of the cells in the target area. If the current geometric center point $q_{i}$ that needs to be cruised contains the centroid region *g*, then the algorithm checks whether the next center point $q_{i+1}$ ’s cell also contains the centroid region *g*. If it does, the algorithm continues to check the number of centroid regions in the cell of center point $q_{i+2}$, until the cell of center point $q_{i+n}$ does not contain the centroid region *g*. At that point, the algorithm finds the shortest path that passes through all centroid regions g between the current center point $q_{i}$ and center point $q_{i+n}$, and repeats this process until all waypoints are covered in the target area, thus obtaining the UAV’s cruising trajectory $\left\{q\left(t\right)\right\}$. The pseudo-code of the algorithm is shown in Algorithm 1.
**Algorithm 1** Cruise communication coverage algorithm based on internal helix**Input:** cell center location, key area location, UAV coverage radius *R***Output:** UAV cruise track q(t)**The process:**Set the center point of the cell closest to the origin of coordinates in the target region as the initial waypoint $q_{0}$; The UAV looks for the geometric center point of the nearest cell as the next waypoint $q_{1}$ in the internal spiral direction clockwise or counterclockwise.If the cell of the next geometric center point $q_{i}$ contains key regions *g*, the number of key regions $s_{j}$ in the cell is calculated; Find the next nearest central point $q_{i+1}$ and judge the number of key regions $s_{j+1}$ in the cell of the central point $q_{i+1}$ until there is no key region in the cell of the next nearest central point $q_{i+n}$.The shortest path algorithm is adopted to calculate the shortest path from the current central point $q_{i}$ through the key area *g* to the central point $q_{i+n}$.Otherwise, directly take the center point of the next cell as the current optimal waypoint.Repeat Steps 2–5.Use a straight line to connect each waypoint in the order of access.Return the cruise track q(t) of the UAV.

As the UAV’s flight trajectory $\left\{q\left(t\right)\right\}$ is a continuous-time function, and the completion time $T_{t}$ of the mission is unknown, a path discretization method proposed by Zeng Yong et al. [37] is adopted to obtain a form with a finite number of optimization variables that is easier to handle. It is worth noting that trajectory and path are two different terms. Path refers to the route followed by the UAV, which includes all the positions on the UAV’s trajectory without involving the time dimension. On the other hand, trajectory includes both the path and the instantaneous velocity of movement along the path, and it involves the time dimension.

When the UAV is cruising to provide communication coverage over the target area, it needs to complete the coverage of all the geometric center points and key regions. Therefore, the path between two adjacent waypoints can be considered as a path segment, and the UAV’s flight trajectory can be discretized into path segments. The expression for a path segment can be obtained as follows:

(23) $$\left|\right|q_{m+1}\left(t\right)-q_{m}\left(t\right)\left|\right|\leq \Delta {}_{\max},\forall m\in M,M=\left\{1,2,\ldots M\right\}$$

where $\Delta {}_{\max}$ is a properly chosen value; and Tm=qm+1t−qmtqm+1t−qmtv′v′ represents the UAV’s flight time within the discretized path segment. By discretizing the path, the UAV’s flight trajectory $q\left(t\right)$ can be represented by M+2 waypoints ${\left\{q_{m}\left(t\right)\right\}}_{m=0}^{M+1}$, and the time spent by the UAV in each path segment can be represented by ${\left\{T_{m}\right\}}_{m=0}^{M+1}$. The energy consumed by the UAV during the cruising process is represented by Equation (24).

(24) $$E_{f}=P_{f}\left(v^{\prime }\right)\sum_{m=0}^{M}T_{m}$$

The energy consumption of the UAV during hovering-assisted communication in a critical area can be expressed as follows:

(25) $$E_{h}=P_{h}\sum_{g=0}^{G}T_{h}$$

where $T_{h}$ represents the time spent hovering in the target area. The energy consumed during the UAV flight or hovering process related to communication is represented as follows:

(26) $$E_{c}=P_{c}\left(\sum_{m=0}^{M}T_{m}+\sum_{g=0}^{G}T_{h}\right)$$

The optimized energy consumption for UAV cruise communication coverage can be obtained by combining Equations (24)–(26):

(27) $$\begin{array}{cc} & E_{t}=E_{f}+E_{h}+E_{c}\\ & \;\;\;\;\;\;=P_{f}\left(v^{\prime }\right)\sum_{m=0}^{M}T_{m}+P_{h}\sum_{g=0}^{G}T_{h}+P_{c}\left(\sum_{m=0}^{M}T_{m}+\sum_{g=0}^{G}T_{h}\right) \end{array}$$

Therefore, according to Equation (27), the energy consumption during UAV cruise communication coverage includes the energy consumption during UAV flight $E_{f}$, the energy consumption during UAV hovering $E_{h}$, and the energy consumption during UAV-assisted communication $E_{c}$.

## 5. Simulation Experiment and Result Analysis

### 5.1. Simulation Setting

To thoroughly validate the performance of the proposed UAV cruise communication coverage strategy, this section conducts the performance evaluations of the strategy under different coverage algorithms, including the UAV multicast coverage path planning algorithm (MCPA) [41] and the traditional inward spiral coverage algorithm (Spiral). In the experimental process, the simulated environment is set as a rectangular target area after a disaster, with the dimensions of the target area being L×W = 3.5 (km) ×3 (km). Users in the target area are randomly distributed, with the critical area represented by the shaded region in Figure 2. When the UAV cruises to the critical area, it hovers and communicates for a period of time $T_{h}$ according to the hover-communication protocol, with the UAV’s communication-related power being denoted as $P_{c}=5$ (W).

The UAV cruises over the target area at an optimal constant speed to satisfy the communication needs of ground users, using the IS-CCC algorithm for UAV trajectory planning. Additionally, the UAV starts cruising from any position near the boundary and completes all waypoints, then returns to the starting point in a straight line to replenish energy if necessary. The proposed strategy is simulated and verified in the MATLAB simulation platform, with specific simulation parameters shown in Table 2 as follows.

### 5.2. Experimental Results and Analysis

In the analysis of the simulation results, the flight trajectory of the UAV for cruising and communicating over the target area is first displayed. Then, the total energy consumption and cruising efficiency of the UAV for covering the target area are analyzed. Finally, the relationship between the UAV energy consumption and the number of priority areas is analyzed and compared with other algorithms.

**Figure 4:**
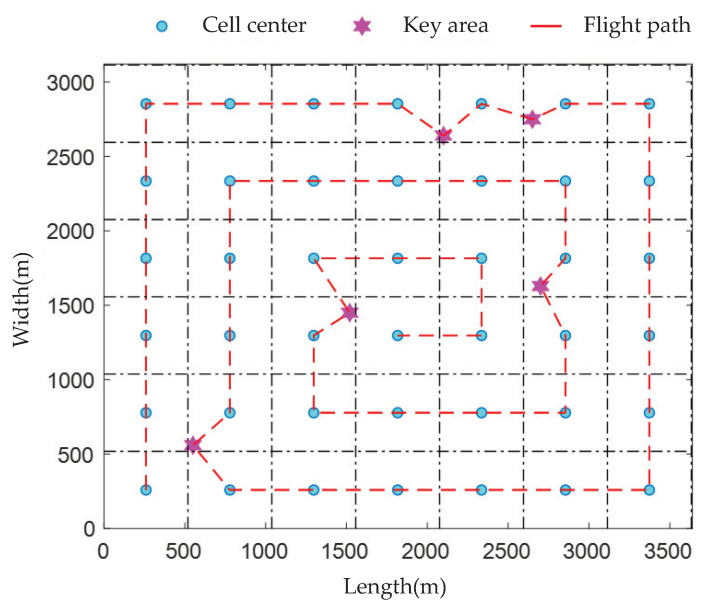
Trajectory planning based on the IS-CCC algorithm.

As shown in Figure 4, the trajectory planning for achieving the full coverage of the target area using the IS-CCC algorithm is demonstrated under the grid-based representation, with a UAV flight speed of 10 m/s and a minimum data rate of Q=15 Mbit to satisfy the communication needs of ground users. Each grid cell has a size of 519 m × 519 m, and there are five priority areas denoted by coordinates $x_{k}$ = [550, 1520, 2700, 2100, 2650] and $y_{k}$ = [560, 1450, 1630, 2640, 2750]. From the figure, it can be observed that the UAV starts from the position closest to the origin and cruises in a spiral manner to cover all waypoints, optimizing energy consumption for covering priority areas until all waypoints are covered to achieve the mission objective. It can be observed from the figure that, when a priority area exists in any grid cell, the UAV follows the shortest path from the previous cell to the next cell and hovers to cover the priority area.

**Figure 5:**
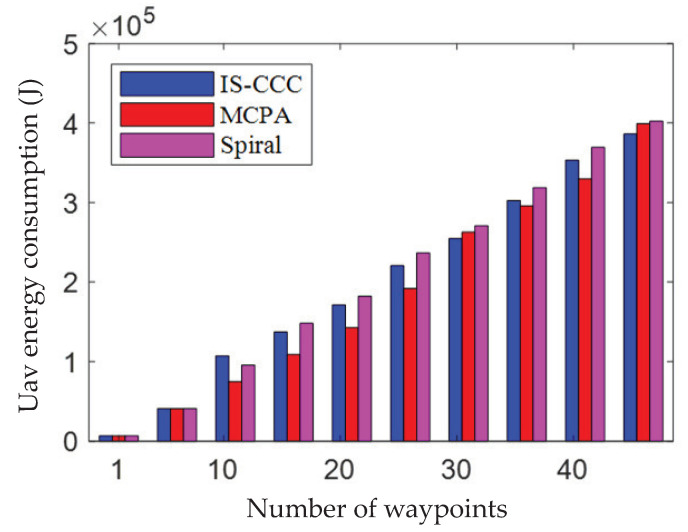
Relationship between the UAV energy consumption and the number of waypoints.

As shown in Figure 5, the impact of the number of waypoints on UAV energy consumption is demonstrated when the number of priority areas is set to 5, and it is compared with the traditional Spiral algorithm and MCPA algorithm. From the figure, it can be seen that the IS-CCC algorithm requires the lowest energy consumption as the number of waypoints increases. Among them, the traditional Spiral algorithm consumes the most energy, reaching $4.02\times 10^{5}$ (J). The MCPA algorithm consumes intermediate energy, approximately $3.99\times 10^{5}$ (J); while the proposed IS-CCC algorithm consumes the least energy, only $3.86\times 10^{5}$ (J). Compared to the Spiral algorithm, the energy consumption of the IS-CCC algorithm is reduced by $0.16\times 10^{5}$ (J). In addition, there is a sudden increase in energy consumption for all three algorithms in the figure. This is because, when the UAV cruises to a priority area, a hover-communication protocol is used for communication, and the energy consumption during hovering and communication at that waypoint causes a sudden increase in UAV energy consumption.

**Figure 6:**
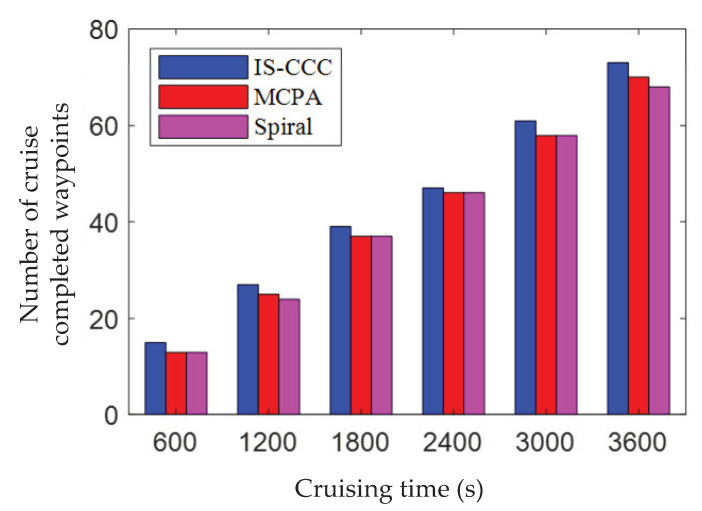
UAV cruise communication coverage efficiency.

Figure 6 illustrates the cruising efficiency of UAVs for communication coverage in the target area, with a mission time of $T_{t}$ = 1 h. From the figure, it can be observed that, when the number of priority areas is set to 5, the IS-CCC algorithm has the highest cruising efficiency with a total of 73 waypoints, while the MCPA algorithm has 70 waypoints and the Spiral algorithm has the fewest waypoints at 68. The IS-CCC algorithm proposed in this paper cruises three more waypoints compared to the MCPA algorithm, and five more waypoints compared to the Spiral algorithm. In addition, the cruising efficiency of the Spiral algorithm is relatively low, which may be due to the fact that the Spiral algorithm tends to maximize local cruising efficiency during the cruising process, which could result in a lower overall cruising efficiency.

**Figure 7:**
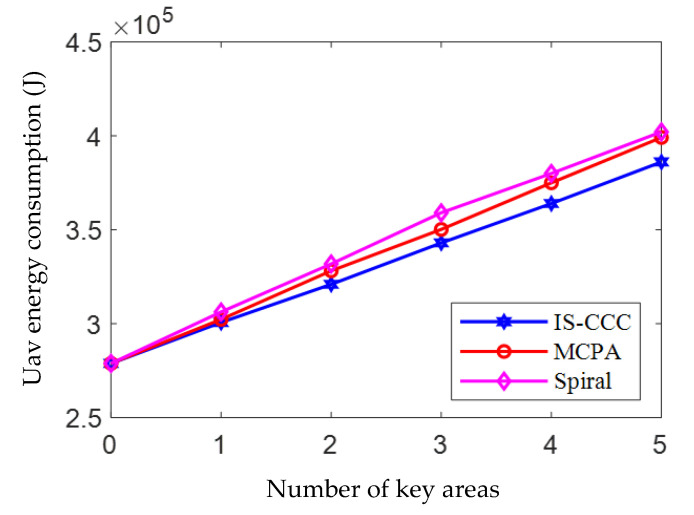
The relationship between UAV energy consumption and the number of key areas.

Figure 7 presents the impact of the number of priority areas on the UAV flight energy consumption. From the figure, it can be observed that, as the number of priority areas increases, the energy consumption of UAVs also increases. Furthermore, the IS-CCC algorithm consumes the least amount of energy compared to the other two algorithms. For example, when the number of priority areas is set to 4, the energy consumption of the IS-CCC algorithm is $3.63\times 10^{5}$ (J), which is $0.12\times 10^{5}$ (J) lower than the energy consumption of the Spiral algorithm, and $0.17\times 10^{5}$ (J) lower than the energy consumption of the MCPA algorithm. When there are no priority areas (i.e., the number of priority areas is 0), the energy consumption of the three algorithms is the same. This phenomenon occurs because, when there are no priority areas, the UAV’s cruising trajectory is the same, resulting in the same energy consumption for all algorithms.

In addition, from the figure, it can be observed that, as the number of priority areas increases, the energy consumption curve fluctuates and rises rather than uniformly increases. This is because the optimal cruising trajectory for the three algorithms differs due to the varying positions of the priority areas, resulting in differences in UAV propulsion energy consumption.

**Figure 8:**
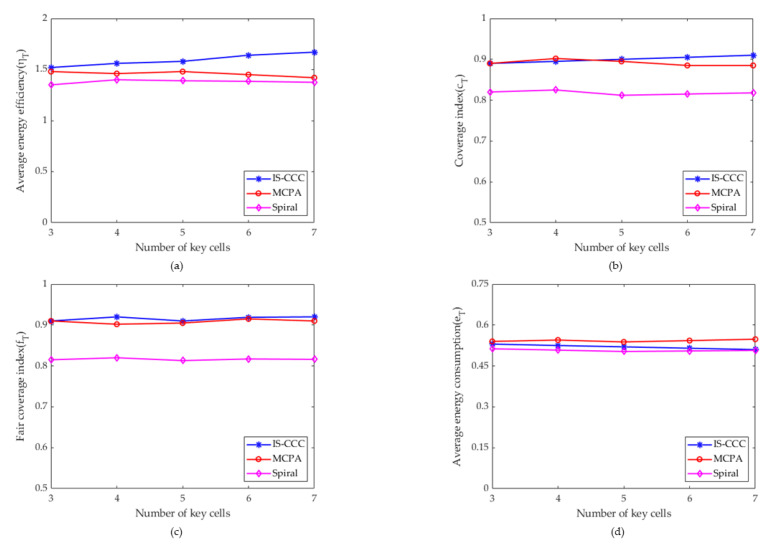
The influence of the number of key cells on average energy efficiency, coverage index, fair coverage index, and average energy consumption. (**a**) The relationship between average energy efficiency and the number of key cells. (**b**) The relationship between the coverage index and the number of key cells. (**c**) The relationship between the fair coverage index and the number of key cells. (**d**) The relationship between average energy consumption and the number of key cells.

Figure 8 shows the impact of the number of key cells on the average energy efficiency, coverage index, fairness coverage index, and average energy consumption. It can be observed that the IS-CCC strategy outperforms the other two strategies in all four evaluation metrics.

First, the effect of the number of key cells on the average energy efficiency was compared, as shown in Figure 8a. The average energy efficiency of the IS-CCC strategy slowly increases with the increase in the number of key cells, while the average energy efficiency of the other two algorithms remains basically stable. This is because with the increase in the number of key cells, the UAVs in the IS-CCC strategy reduce the energy and flight time consumption by flying and increase the hovering time to improve the coverage rate index, resulting in a slow increase in the average energy efficiency. In Figure 8b, as the number of key cells increases, the coverage index of the IS-CCC strategy continues to increase, while those of the MCPA and Spiral strategies remain basically unchanged. This is because, as the number of key cells increases, the UAVs in the IS-CCC strategy reduce the time of flight consumption and increase the hovering time, which leads to an increase in the coverage index. In Figure 8c, it can be observed that the fairness coverage index of IS-CCC, MCPA, and Spiral strategies remains almost unchanged with an increase in the number of critical cells. This is because the increase in the number of critical cells has little effect on the fairness coverage strategy of UAVs, and therefore, the fairness coverage index remains constant. In Figure 8d, the average energy consumption of all strategies remains almost unchanged as the number of key cells increases. This is because, with the increase in key cell numbers, the control strategies of the UAVs remain unchanged, and therefore, the average energy efficiency remains basically unchanged.

## 6. Conclusions

In this paper, aiming at the communication coverage problem for complex user distribution scenarios in an emergency environment, we proposed a UAV-based cruise communication coverage strategy for complex user distribution scenarios. The strategy utilizes UAVs to assist in post-disaster communication through cruise communication mode. Firstly, the minimum transmission rate that meets the user’s communication requirements is set, and the post-disaster target area is divided into multiple grid cells. Then, based on the characteristics of user distribution, UAVs are used to assist user communication in ordinary cells through a fly-communicate mode, and in priority areas through a hover-communicate mode. To optimize energy consumption during UAV cruise communication and improve the efficiency of UAV-assisted communication, the strategy proposes the IS-CCC algorithm to plan UAV flight paths by optimizing UAV flight speeds, achieving energy-efficient UAV cruise communication while ensuring the recovery of ground user communication. The simulation results show that the proposed strategy can effectively achieve UAV communication coverage in disaster areas under complex user distribution scenarios, and it outperforms other strategies in terms of coverage rate index, fair coverage index, and average energy efficiency.

## Figures and Tables

**Table 1:** Symbolic representation of power consumption for rotary-wing UAV.

Symbol	Physical Meaning
$P_{0}$	Blade profile power (W)
$P_{i}$	Induced power (W)
$U_{tip}$	Tip velocity of rotor blade (m/s)
$v_{0}$	Average rotor-induced velocity at hover (m/s)
$d_{0}$	Airframe resistance ratio
ρ	Air density (kg/m${}^{3}$)
*s*	Rotor firmness
*A*	Rotor disk area (m${}^{2}$)
δ	Contour resistance coefficient
Ω	Blade angular velocity (rad/s)
*R*	Rotor radius (m)
*k*	Rotor thrust increment correction coefficient of induced power
*W*	UAV weight (N)

**Table 2:** Simulation parameters.

Parameter	Value
Blade profile power $\left(P_{0}\right)$	80 W
Induced power $\left(P_{i}\right)$	88.5 W
Tip velocity of rotor blade $\left(U_{tip}\right)$	120 m/s
Average rotor induced velocity at hover $\left(v_{0}\right)$	4.03 m/s
Airframe resistance ratio $\left(d_{0}\right)$	0.6
Air density (ρ)	1.225 kg/m${}^{3}$
Rotor firmness (s)	0.05
Rotor disk area (A)	0.503 m${}^{2}$
Contour resistance coefficient (δ)	0.012
Blade angular velocity (Ω)	300 rad/s
Rotor radius (R)	0.4 m
Rotor thrust increment correction coefficient of induced power (k)	0.1
UAV weight (W)	20 N
Launch power of UAV $\left(P_{U}\right)$	37 dBm
System available channel set (L)	50
Channel power gain at 1 m (β)	−75 dB
Noise power $\left(N_{0}\right)$	−104 dBm
UAV flying altitude (H)	100 m
UAV coverage radius (R)	300 m
System bandwidth (B)	1 MHz
Data information threshold (Q)	15 Mbit
UAV hovering time $\left(T_{h}\right)$	120 s
Communication-related power $\left(P_{t}\right)$	5 W

## Data Availability

Not applicable.
